# Posterior Tibial Tendon Dysfunction: An Overview

**DOI:** 10.2174/1874325001711010714

**Published:** 2017-07-31

**Authors:** Samuel Ka-Kin Ling, Tun Hing Lui

**Affiliations:** Department of Orthopaedics and Traumatology, North District Hospital, Hong Kong SAR

**Keywords:** Flatfoot, Adult acquired flatfoot deformity, Posterior tibial tendon dysfunction, Posterior tibial tendon insufficiency, Posterior tibial tendon, Pes planus, Calcaneovalgus, Foot arch

## Abstract

**Background:**

Adult acquired flatfoot deformity is a commonly seen condition with a large clinical spectrum. It ranges from asymptomatic subjects to severely disabled arthritic patients. Posterior tibialis tendon dysfunction is a common cause of adult acquired flatfoot deformity.

**Methods:**

This article systematically reviews the published literature from books and journals that were either originally written or later translated into the English language regarding the subject of posterior tibialis tendon dysfunction.

**Results:**

Posterior tibialis tendon dysfunction is a primary soft tissue tendinopathy of the posterior tibialis that leads to altered foot biomechanics. Although the natural history of posterior tibialis tendon dysfunction is not fully known, it has mostly been agreed that it is a progressive disorder. While clinical examination is important in diagnosing adult acquired flat-feet; further investigation is often required to delineate the different aetiologies and stage of the disease. The literature describes many different management choices for the different stages of posterior tibialis tendon dysfunction.

**Conclusion:**

Because of the wide range of symptom and deformity severity, surgical reconstruction is based on a-la-carte. The consensus is that a plethora of reconstructive options needs to be available and the list of procedures should be tailored to tackle the different symptoms, especially when managing complex multi-planar reconstructions.

## 1 INTRODUCTION

Flatfoot is a clinical entity which involves the flattening of the medial foot arch; it comprises of a large clinical spectrum with multiple aetiologies [1-4]. It is often differentiated into 2 major groups according to the age of presentation; this is because the congenital and adult types have distinct and differing aetiologies.

The overall prevalence of flatfeet has been reported to range from 5-15% in the general population; roughly 7-15% of these patients are symptomatic and will seek medical attention [5-8]. Rough calculations show that this figure equates to roughly 1 out of 100 people in the entire population seeking medical management for symptomatic flatfeet. 1% prevalence is not a high number but it does comprise a significant portion of work in a sub-specialised foot and ankle clinic. However, these statistics are difficult to interpret since the exact definition and diagnostic criteria of flatfeet are unclear. While most experts agree that late stage symptomatic cases are relatively clear-cut; the prevalence of asymptomatic flatfoot is still under intense debate.

The fundamental, yet unanswered question is: what is the normal of foot arch? Multiple investigators have used various innovative methods and state-of-the-art technologies to measure and quantify foot arches [9-11], but none have established a standard normative value or proven that their method is superior to the others. As of the current knowledge, there is no gold standard measurement tool or universally accepted normal variation range. Radiological parameters may provide a framework for objective measurement; but as with the clinical parameters, none have been fully validated and the coveted normal values are not yet agreed upon [12-14].

Because of the wide and varied aetiologies, this article will take a pragmatic approach and focus solely on adult acquired flatfoot deformity due to posterior tibial tendon dysfunction/insufficiency. It is the most commonly seen flatfoot condition which entails a large clinical spectrum ranging from pure soft tissue tendinopathy to full-fledged arthropathy [15, 16].

## 2 ANATOMY

### 2.1 Course

The posterior tibialis muscle originates from the posterior proximal tibia, interosseous membrane and proximal fibula; it is in the deep posterior compartment of the leg and turns at the medial malleolus, changing its line of pull from the vertical one to the horizontal one [17, 18]. The myotendinous junction is situated at the distal third of the leg and only the tendinous portion turns at the medial malleolus; anterior subluxation is prevented by a flexor retinaculum which binds the tendon into the shallow groove at the medial malleolus. It has a broad insertion into the plantar medial midfoot; with a major portion inserting into the navicular tuberosity, another into the sustentaculum tali, and the remaining portion inserting into the entire plantar midfoot except for the 5th metatarsal.

### 2.2 Vascularity

The posterior tibialis is relatively prone to develop insufficiency due to its relatively unique tendon sheath. A generic tendon sheath usually comprises of 3 layers: an outer parietal layer which provides a fibro-osseous canal, a middle mesotenon layer which usually contains the blood supply, and a visceral layer immediately surrounding the tendon. In the posterior tibialis, the mesotenon, which usually contains the vascular supply, is incomplete; this jeopardises its overall blood supply [19]. The vascularity is good at the musculotendinous junction (with supplies from the posterior tibial artery) and the osseous-tendon junction (with supplies from the periosteal branches of the medial plantar artery which originates from the posterior tibial artery). But the middle watershed portion between these 2 points is termed as the zone of hypovascularity; some have documented this zone to roughly span from 14mm from the navicular insertion up to the medial malleolus. It is in this hypovascular zone, in which, tendinopathy is significantly observed [20].

### 2.3 Function

Due to its anatomical course, the axis of action of the posterior tibialis is posterior to the tibial-talar (ankle) joint and thus functions as a plantar flexor of the ankle. At the subtalar joint; its axis is medial and inferior, thus it acts as an adductor and supinator; which results in the combined motion of midfoot inversion.

Aside from its active motion, the posterior tibialis also helps maintain the foot arches. Generally, structures which provide arch support can be divided into static and dynamic types. Structures which provide static support include the bone as weFIGll as the plantar ligaments.

Various theories including the truss and beam theories have been proposed to explain their mechanism of action. The plantar ligaments include the long and short plantar ligaments, calcaneo-navicular (spring) ligament, and the bifurcate ligament. All these ligaments are insertion points of the posterior tibialis muscle; thus the intrinsic tension in the posterior tibialis muscle helps maintain integral tightness in these ligaments. If the tension from the posterior tibialis is lost; it is not uncommon to see associated failure in these plantar ligaments, especially the calcaneo-navicular ligament [21]. Aside from the static arch restraints; the posterior tibialis and intrinsic muscles provide a dynamic maintenance of foot arches.

In the initial stance phase; the posterior tibialis muscles fires to stabilise the hindfoot from valgus and decelerates midfoot pronation. At mid-stance, it initiates inversion at the subtalar joint, effectively locking the midfoot articulation which provides the required foot rigidity for effective toe-off. Contraction of the posterior tibialis adducts and plantarflexes the navicular on the talar head; buttressing the medial arch against collapse. As stated, the posterior tibialis inserts into the plantar ligaments; thus a contraction in this tendon leads to increased tension in the plantar ligaments which pulls the cuboid medially. More importantly, via the link of the calcaneo-cuboid ligaments; the cuboid also pulls the calcaneum medially as an additional medial support for the talus [22].

### 2.4 Pathophysiology

The natural history of posterior tibialis tendon dysfunction is not fully known, but most agree that it is a progressive disorder. Although the exact pathophysiology is not well understood, this primary soft tissue tendinopathy in the posterior tibialis leads to altered foot biomechanics. This results in secondary reactive changes including subsequent ligament and tendon attenuation, reactive hallux valgus due to transversely shifted weight bearing and secondary osteoarthritis due to instability. The commonly used staging system developed by Johnson and Strom and subsequently modified by Bluman and Myerson splits the disease into 4 different clinical stages correlating to different pathoanatomies within the disease spectrum [23, 24].

The first two stages have only soft tissue problems while the third and fourth stages have resulted in associated joint arthritis. Stage 1 is isolated posterior tibialis tendinopathy without a significant change in tendon length. The second stage involves permanent changes in the posterior tibialis tendon which can be elongated in stage 2a and attenuated in the more advanced stage 2b. Stage 3 is defined when features of subtalar arthritis occur, and stage 4 signifies involvement of the ankle joint. While clinical examination is important in diagnosing adult acquired flatfeet; further investigation is often required to delineate the different aetiologies and stage of the disease.

### 2.5 Investigations

Investigations for flat feet include radiological imaging as well as various forms of foot pressure analysis.

The most common is the X-ray; which should be weight-bearing images that show the usual alignment in the physiological stance phase. Aside from ruling out other flatfoot aetiologies such as tarsal coalition; X-rays also help to quantify the degree of deformity. A host of radiological parameters has been proposed to quantify foot arches objectively. The talo-metatarsal angle in the lateral standing view of the foot is the angle between the lines bisecting the talus and 1st metatarsal shaft. A negative talo-metatarsal angle signifies a break in the midfoot, which is commonly noticed when the foot arch collapses. Normative values in the lateral talo-metatarsal ankle has been quoted to range from -4 - 4 degrees [25, 26]. The cuneiform height is another lesser known method of measurement which has been reported to be more accurate and has less inter-observer error compared to the talo-metatarsal angle measurement. The height difference between the middle cuneiform base and the 5th metatarsal base is measured; with normative values suggested at around 18mm. However, the available published evidence is inconclusive. On the dorsal-plantar projection; the talo-calcaneal angle is situated between the mid-axial line of the talus and mid-axial line of the calcaneum. The mid-talar line should pass at the lateral 1st web space while the mid-calcaneal line should pass the 4th metatarsal. The angle between these 2 lines is the talo-calcaneal angle which is reported to range from 25-40 degrees. Another measurement is the talo-navicular coverage which consists of two lines, one bisecting the talus and the other bisecting the navicular. It measures the articular coverage of the navicular on the talus and is usually 35-50 degrees; anything more than 50 degrees signifies some hindfoot valgus deformity.

Aside from X-rays, ultrasound is helpful in evaluating the posterior tibialis tendon. The benefit of ultrasound is that it allows real-time dynamic assessment of the tendon, but it is operator dependent [27]. It has been reported that a tendon width of more than 6mm is suggestive of tenosynovitis; especially if associated with the presence of a ‘target sign’ which is a hypo-echoic rim of fluid surrounding the hyper-echoic tendon substance. Some have reported that a tendon substance size of >4mm or a sheath size of >7mm is representative of tenosynovitis in the posterior tibialis tendon.

More advanced imaging modalities such as CT has also been suggested, but its greatest use is for the exclusion of bony flatfeet aetiologies such as tarsal coalition. For adult acquired flatfeet; MRI is preferred over CT because it provides better visualisation of soft tissue structures. Its sensitivity is reported to be 95% compared to 90% via CT, and it has a higher chance to accurately diagnose longitudinally split tears [28]. An artefact known as the ‘magic angle effect’ is seen on T1 weighted MRI images; it occurs at the turning point of the posterior tibialis tendon which is situated near the medial malleolus. This artefact gives a false image similar to a ruptured tendon; however, it is easily differentiated from a true tendon rupture by obtaining a true axial plane image perpendicular to the tendon at this level. In addition, T2 weighted images do not have this magic angle effect artefact [29]. T1 weighted MRI shows a thickened posterior tibialis tendon substance and sheath, especially compared with the adjacent flexor hallucis longus tendon. T2 images also allow visualisation of the hyper-intense peri-tendinous collection; a thickness of >2mm is considered abnormal.

Aside from anatomical imaging; foot pressure analysis are also important investigations for objective assessment of foot arches. Various footprint and foot pressure analysis are available such as the Harris mat, with different measurements parameters like the Staheli index [30, 31]. These measurements all give information on foot arches, but these parameters have not been fully validated and tested [32].

### 2.6 Stage 1 Posterior Tibial Tendon Dysfunction

In stage 1 disease with pure tendinopathy without significant elongation or rupture, the symptoms are usually pain over the medial foot as well as decreased endurance. On physical examination, there is evidence of inflammation such as erythema, swelling, increased temperature and localised tenderness over the posterior tibialis tendon. The medial foot arch is usually maintained and the overall foot alignment is normal. Posterior tibialis muscle power is only subtly decreased with a grossly normal single leg heel raise; however, the decreased endurance results in failure to perform multiple successive single leg heel raises.

X-rays are usually unremarkable, but ultrasound and MRI reveal evidence of tenosynovitis such as the target sign.

Management begins with non-surgical options. Immobilisation and rest with a plaster cast are the traditional methods which provide good protection; assuming it is tolerated by the patient. Other devices such as a controlled ankle movement walker boot are also solid choices and have less muscular atrophy compared to plaster casting. These are often the preferred choice since allowing partial weight bearing walking has also been suggested to stimulate better collagen organisation [33]. Orthotics have also been widely used to eliminate forefoot pronation and rest the posterior tibialis tendon. Many different forms have been described such as the UCBL / Arizona brace/ ankle foot orthosis and the simpler medial heel wedge.

In additional to immobilisation; pharmacological management is also important. Analgesics such as various NSAID in oral and injectable forms have been used to alleviate the pain. Use of corticosteroids is controversial because although in theory, they can help subdue the inflammation, but it has also been reported that they create unwanted microvascular attenuation which further jeopardises the tendon circulation. Other novel therapies such as platelet rich plasma might be beneficial, but published evidence is lacking [34].

Physiotherapy also helps in two regards; first, it aids in decreasing the swelling in acute inflammation. Once the initial inflammation has subsided, physiotherapy to strengthen the posterior tibialis can be performed. Methods to decrease the initial swelling include cryotherapy and iontophoresis. Iontophoresis is a procedure which transmits localised dexamethasone via an electrical current deep to the skin into the inflamed tendon. As stated above, some have raised concerns about steroids jeopardising the tendon’s circulation, but results are inconclusive. Heat and ultrasound are usually not used in the acute phase as they exacerbate inflammation and worsen the clinical inflammation. Once the inflammation has subdued, strengthening of the posterior tibialis can be performed by simple repetitions of the single leg heel raise, or with the addition of external weights and devices such as resistance bands [5].

Surgery is usually reserved for the recalcitrant cases or in cases associated with inflammatory arthropathies as it has been shown they require more aggressive management. In stage 1 PTTD, the mainstay of surgical treatment is synovectomy since literature from the 1950s and 1960s by the likes of Fowler and Langenskiold have documented improvement [35-37]. Minimally invasive techniques for tendon synovectomy have been published with fairly good outcomes. Endoscopic synovectomy of the posterior tibialis tendon is performed using a 2.7mm arthroscope with the visualisation portal near its navicular insertion and a more proximal working portal for synovectomy [35]. However, the traditional open procedure with a roughly 5cm wound along the tendon course is still the standard. An important precaution is to have a backup plan of proceeding to more advanced repair/reconstruction if significant intra-substance degeneration is found intra-operatively. This is important because although pre-operative investigations have acceptable accuracy, some more advanced stages are still missed [38].

### 2.7 Stage 2 Posterior Tibial Tendon Dysfunction

Stage 2 posterior tibial tendon dysfunction is a wider spectrum of disease compared to stage 1 and is more commonly seen in the orthopaedic setting. It has irreversible tendon changes with elongation in stage 2a and progressing to tendon rupture in stage 2b [39].

Symptoms are similar to stage 1 with predominantly inflammatory discomfort. This is sometimes associated with decreased strength in the posterior tibialis which usually manifests as decreased endurance in the earlier stages. Physical examination shows the inflammatory signs of swelling, erythema and tenderness over the course of the posterior tibialis. The hindfoot is often in valgus which is flexible; the midfoot medial arch is flattened but returns upon single leg raise and the forefoot may be abducted and supinated. Stage 2b disease will also have a larger element of lateral heel pain compared to 2a; especially over the sinus tarsi region.

Investigations would reveal a midfoot break at the talo-metatarsal angle, the talo-calcaneal angle and a decreased cuneiform height. Normative values of the talometartarsal angle were stipulated to range from -4°-4° by Gould; while Saltzman commented that a talocalcaneal angle of <12° is considered normal. The cuneiform height is measured by identifying the difference between the middle cuneiform and the 5th metatarsal base. Other useful imaging modalities include ultrasound and MRI. In stage 2a disease, ultrasound will show similar tenosynovitis changes as seen in stage 1; the degenerative tendons turns from being hyper-echoic to hypo-echoic. Stage 2b disease may show the empty groove sign, which signifies a ruptured tendon gap. MRI can reveal an elongated and thinned tendon; because of this elongation, it is reportedly less hypertrophic when compared to the tendon size in stage 1 disease. On comparison with the adjacent flexor digitorum longus tendon, the posterior tibialis tendon may be even smaller in diameter.

Surgical management is more complex as the elongated or ruptured tendon requires more than a simple synovectomy; the general principle is to have an arsenal of reconstruction options to perform as required. Since stage 2 disease, by definition, should not have any associated arthropathy; soft tissue reconstructive procedures are preferred over bone and joint reconstruction techniques. A tendon transfer is often the management of choice with some reporting on the use of the flexor hallucis longus while others preferring the flexor digitorum longus. The flexor digitorum longus is directly adjacent to the posterior tibialis tendon and is easy to manipulate as it has the same line of pull. In addition, it is an in-phase muscle at mid-stance and allows to maintain hallux flexion by sparing the flexor hallucis longus [40]. Lesser toe flexion is also partially retained since the flexor hallucis longus has attachments on the flexor digitorum longus tendons. However, the flexor digitorum longus is comparatively thin and small, thus the transfer of the flexor hallucis longus is often preferred due to its more matched size to the posterior tibialis. In conjunction with the tendon transfer, calcaneonavicular ligament plication is often performed in the same instance [41, 42]. This can be performed via a traditional open approach, or an endoscopically assisted approach [43-45]. Endoscopic-assisted posterior tibialis reconstruction can be performed by using the medial half of the anterior tibialis tendon as a primary transfer which is augmented by the flexor digitorum longus. This is done by first establishing a posterior tibialis tendon portal near its navicular insertion for visualisation, and then a working portal at the proximal end of the tendinopathy; synovectomy is performed if necessary. The anterior tibialis is visualised with a portal near its insertion, and the tendon is traced until the myotendinous junction is identified; the myotendinous junction is the site of the proximal portal and the medial half of the anterior tibialis tendon is harvested down to the insertion. This graft is transferred to the posterior tibialis and anchored proximal to the diseased tendon; flexor digitorum longus tendoscopy is performed and then it is augmented onto the posterior tibialis tendon. This construct is then protected with a subtalar arthroereisis which is inserted under fluoroscopic assistance [41, 43].

Regardless of the method of reconstruction; a protective mechanism is required to prevent premature re-elongation or re-rupture of the reconstructed posterior tibialis. The most commonly used protection is some form of calcaneal osteotomy; this corrects calcaneal-valgus and shifts the line of pull of the Achilles tendon away from being a deforming force. These osteotomies are increasingly performed via minimally invasive methods using percutaneous techniques [46]. The medial slide calcaneal osteotomy shifts the mechanical pull of the Achilles tendon medially, offloading the transferred flexor digitorum longus [47] and shifts the weight bearing axis of the heel closer to the weight bearing axis of the tibia, effectively decreasing the valgus causing force.

Other commonly used calcaneal osteotomies include the double calcaneal osteotomy and the percutaneous osteotomy [48]. Aside from calcaneal osteotomies; the option of subtalar arthroereisis is gaining in popularity to protect the reconstruction. Originally mostly used in the paediatric population, arthroereisis has been adopted for use the adult acquired flatfoot deformity with good results. It is a device which stabilises the subtalar joint and prevents excessive hindfoot valgus by supporting the talus and preventing its plantar-medial displacement [49-51].

In addition to the previous procedures, calcaneo-navicular ligament reconstruction and repair has also been reported to be a useful adjunct. The ligament is split into 3 major bands, the superior-medial, medial plantar oblique and the plantar inferior complex [52, 53]. Reconstruction of the calcaneo-navicular ligament with a peroneal longus graft can be performed with acceptable results; however high-level evidence with direct comparisons is lacking [54].

The lateral column of the foot is sometimes shortened and a lateral column lengthening procedure can be performed such as the Evans lateral calcaneal opening wedge osteotomy. If there is significant lateral impingement or when the talo-navicular joint is abducted, a step cut calcaneal lateral column lengthening osteotomy can also be a good choice; however one should be careful to avoid fusion of the subtalar and talo-navicular joints.

At the forefoot, the aim is to maintain the tripod mechanism. If there is a fixed dorsiflexion of the first ray, a plantar-flexion opening wedge osteotomy of the medial cuneiform is the adjunct procedure. This cotton osteotomy is usually preferred over corrective 1st metatarsal-cuneiform fusion because it is easier to perform; however, the 1st metatarsal-cuneiform fusion has a better corrective power and can be used in cases with a more severe deformity [55, 56].

In addition to these reconstructive procedures; some cases require selective arthrodesis. Since stage 2 disease does not have significant joint arthritis, the principle is to perform isolated arthrodesis in a minimal number of joints. Results of isolated subtalar arthrodesis, calcaneal-cuboid interposition arthrodesis and medial column arthrodesis have been reported. These isolated arthrodesis can be performed via the traditional open approach, or via minimally invasive techniques [57-60].

Lastly, after the a-la-carte reconstruction of the hindfoot, midfoot and forefoot; if significant equinus is present; a posterior muscle group lengthening procedure such as the gastrocnemius recession should be considered.

### 2.8 Stage 3 Posterior Tibial Tendon Dysfunction

Stage 3 is when the disease has progressed and has evidence of subtalar joint arthropathy and degeneration. Arthritic symptoms will be present in addition to the inflammatory symptoms seen in the earlier stages. Due to the bone and joint pathology; the deformity may be fixed instead of the flexible deformity seen in pure soft tissue conditions. There is decreased subtalar joint motion with a fixed hindfoot valgus, midfoot planus and forefoot abduction with supination.

X-rays is diagnostic and will show evidence of subtalar arthritis; USG and MRI are less useful to guide management in this advanced stage. They do show additional information such as confirming a ruptured tendon and revealing the absence of a normal T1 fat signal along with T2 bone oedema from sub-fibular impingement at the sinus tarsi.

Management usually involves some form of arthrodesis since the joint is already arthritic. As with stage 2, this is usually an a-la-carte reconstruction without a one-size-fits-all procedure. Isolated arthrodesis of the subtalar joint, talo-navicular joint and the calcaneo-cuboid joint have been documented to be useful. Some cases will require triple arthrodesis which can be done via traditional open or minimally invasive approaches. Minimally invasive triple arthrodesis can be done under arthroscopic assistance with the patient supine; subtalar arthroscopy is performed via the anterolateral and middle subtalar portals. Arthroscopic denuding of the cartilage is performed along with lateral capsular and ligamentous release. After the subtalar joint has been adequately prepared, mid-tarsal arthroscopy with the lateral, dorsal-lateral, dorsal-medial and medial portals is performed. The calcaneo-cuboid joint is visualised via the lateral and dorsal-lateral midfoot portals while the talo-navicular joint is approached with the medial, dorsal-medial and dorsal-lateral midfoot portals. Arthroscopic denuding of the articular cartilage is performed under arthroscopic visualisation and transfixion of the foot in the desired alignment is achieved with fluoroscopic guidance. The autologous iliac bone graft can be used as an adjunct procedure to improve fusion rates. As with the stage 2 disease, an additional posterior muscle group lengthening procedure can be performed if there is persistent equinus deformity [61, 62].

### 2.9 Stage 4 Posterior Tibial Tendon Dysfunction

The end stage disease involving degeneration of the tibio-talar joint is less commonly seen. Symptoms are the same as previous stages, with additional arthritic ankle pain and stiffness.

The deformity is fixed with a valgus ankle, calcaneal valgus, midfoot planus and forefoot abduction with supination.

X-rays shows tibio-talar arthritis in addition to subtalar arthritis and planus deformity; as with stage 3 disease; USG and MRI provide minimal additional information at this late stage.

The long-standing ankle instability is often associated with deltoid ligament insufficiency. Since motion in the tibio-talar joint is more important than in the subtalar joint; fusion is often left as a last resort. If arthritis in the tibial-talar joint isn’t too severe; triple arthrodesis in addition to a calcaneal osteotomy with deltoid ligament repair can be considered [63, 64]. Simple repair of the deltoid ligament is not always applicable, which has led to the development of various deltoid reconstruction techniques [65]. One of the most common is the Haddad technique using an autograft such as the semitendinosus tendon; others grafts such as the peroneus longus have also been proposed to reconstruct the deltoid ligament complex [66]. This can be performed using a lateral and medial foot incisions; the peroneus longus is harvested and transferred to the medial side through the sinus tarsi and tarsal canal. This tendon graft is then sutured onto the superficial deltoid ligament and split into 2 limbs. One limb is passed in a figure-of-8 manner through two bone tunnels in the medial malleolus to reconstruct the superficial deltoid ligament, while the other limb is tensioned through an interosseous tunnel at the talus [67].

If the arthritis is too severe, a salvage procedure such as pan-talar fusion can be considered. However, the results are generally poor in these severe end-stage deformity cases [68]. Ankle arthroplasty may be a good solution in the future, but the current technique and prosthesis design are still relatively in its infancy.

## CONCLUSION

Adult acquired flatfoot deformity is a commonly seen condition with a large clinical spectrum. It ranges from asymptomatic subjects to severely disabled arthritic patients. Because of the different severity and multiplanar deformity, surgical reconstruction is based on an a-la-carte basis; a plethora of reconstructive options need to be available when tackling complex reconstructions.

## LIST OF ABBREVIATIONS

● CT: = Computed Tomography
● MRI: = Magnetic Resonance Imaging
● NSAID: = Non-Steroidal Anti-Inflammatory Drugs
● UCBL: = University of California Berkeley Laboratory Shoe Insert
● USG: = Ultrasonography
